# Impact of Warm Vertical Compaction on the Sealing Ability of Calcium Silicate-Based Sealers: A Confocal Microscopic Evaluation

**DOI:** 10.3390/ma14020372

**Published:** 2021-01-14

**Authors:** Diana Eid, Etienne Medioni, Gustavo De-Deus, Issam Khalil, Alfred Naaman, Carla Zogheib

**Affiliations:** 1 Department of Endodontics, Faculty of Dentistry, Saint Joseph University, Beirut BP 17-5208, Lebanon; dianageid@gmail.com (D.E.); issamtkhalil@gmail.com (I.K.); alfrednaaman@gmail.com (A.N.); 2 Micoralis Laboratory EA7354, Faculty of Dentistry, University of Nice Sophia Antipolis, Pôle Odontologie du CHU de NICE, Hopital St. Roch, 06000 Nice, France; etienne.medioni@univ-cotedazur.fr; 3 Department of Endodontics, School of Dentistry, UNIGRANRIO—Universidade Grande Rio, 1.160-jardim 25 DE Agosto, 22061-030 Rio de Janeiro, Brazil; endogus@gmail.com

**Keywords:** calcium silicate, confocal laser scanning microscopy, tubule penetration, warm vertical compaction

## Abstract

The aim of this in vitro study was to evaluate the dentinal tubule penetration of two calcium silicate-based sealers used in warm vertical compaction (WVC) obturation technique in comparison with the single cone (SC) technique by confocal laser scanning microscopy (CLSM). The null hypothesis was that both obturation techniques produced similar sealer penetration depths at 1 and 5 mm from the apex. Forty-four mandibular single-rooted premolars were randomly divided into four equally experimental groups (n = 10) and two control groups (n = 2) according to the type of sealer (Bio-C Angelus, Londrína, PR, Brazil or HiFlow Brasseler, Savannah, GA, USA) with either SC or WVC. The sealers were mixed with a fluorescent dye Rhodamine B (0.1%) to enable the assessment under the CLSM. All the specimens were sectioned horizontally at 1 and 5 mm from the apex. The maximum penetration depth was calculated using the ImageJ Software (ImageJ, NIH). Data were analyzed by Mann–Whitney U and Kruskal–Wallis tests (*p* < 0.05). A significant difference was shown between the four groups at 1 mm (*p* = 0.0116), whereas similar results were observed at 5 mm (*p* = 0.20). WVC allowed better diffusion for both sealers at 1 mm (*p* = 0.01) and 5 mm (*p* = 0.034). The maximum penetration of the Bio-C and HiFlow sealers was more important at 5 mm with the two obturation techniques. Within the limitations of this study, WVC enhanced the penetration of calcium silicate-based sealers into the dentinal tubules in comparison with the SC technique at both levels.

## 1. Introduction

Many obturation techniques have been investigated to seal the root canal system. A three-dimensional obturation is likely to create a fluid-tight seal and to prevent microleakage, which is one of the main causes of endodontic failure [1]. To overcome this challenge, which compromises long-term success, the sealers’ deep penetration into the dentinal tubules is more implicated in producing a sufficient seal to entomb residual bacteria. Moreover, it enhances lateral and vertical sealing by filling spaces and voids [2].

Various types of sealers have been proposed to fill the spaces between the gutta-percha and the canal walls. Ideally, they should create a tight and adequate seal with the core material and dentine to reduce gaps. These requirements are affected by their physicochemical properties and their placement method. Therefore, the selection of an appropriate sealer is mandatory with the selection of the filling obturation technique [3].

Owing to their high biocompatibility, low cytotoxicity, and viscosity, tricalcium silicate-based sealers have aroused renewed interest in relation to improving filling quality [4]. According to the manufacturer, calcium silicate-based sealers such as Endosequence BC (Brasseler USA, Savannah, GA, USA) and iRoot SP (Innovative BioCeramix Inc., Vancouver, BC, Canada) are composed of calcium silicate, calcium phosphate, calcium hydroxide, zirconium oxide, and other agents [5]. They showed effective antimicrobial activity against multiple microorganisms [6]. Furthermore, they revealed a slight volume expansion while setting. These factors improve mechanical retention and chemical bonding to the dentinal walls. A physical barrier to fluids and nutrients is then formed [7]. They are widely indicated with the single cone (SC) technique [8].

However, thermoplasticized gutta-percha shows better canal irregularities in fillings than cold gutta-percha points and promotes the creation of a three-dimensional obturation [9]. Nevertheless, some studies reported that excessive heat might alter the sealers’ properties [10], while others proved the opposite [11,12].

Recently, two new modified sealers HiFlow (Brasseler, Savannah, GA, SA) and Bio-C (Angelus, Londrína, PR, Brazil) have been proposed with warm vertical gutta-percha obturation techniques. According to the manufacturer, HiFlow exhibits a lower viscosity compared to standard BC Sealer when heated and is more radiopaque, making it optimal for warm vertical compaction (WVC). (**Stephen Buchanan**. Warm gutta-percha obturation with BC HiFlow™ Sealer. Endodontic practice US 2018).

To our knowledge, no study has yet evaluated the impact of the warm vertical compaction on the dentinal tubule penetration. The aim of this in vitro study was to evaluate the impact of heat application on the tubular penetration of two silicate-based sealers in comparison with the cold single cone technique using confocal laser scanning microscopy. The null hypothesis tested was that WVC does not enhance both sealers’ penetration compared with the SC technique.

## 2. Materials and Methods

This study was approved by the Ethical Committee of the Saint Joseph University-Beirut (FMD 186, 2018).

### 2.1. Selection of Specimen

Forty-four human mandibular permanent single-rooted premolars were selected in this study. Criteria for the selection of the teeth were one straight canal, no sign of fracture/cracks, absence of internal and external resorption, and no obstruction or calcification within the canal. Two digital radiographs (buccolingual and mesiodistal) were taken to confirm the presence of one canal and the glidepath in each tooth.

### 2.2. Root Canal Treatment

The crowns were removed at 16 mm to standardize the length of all the canals. A standard access preparation was performed for each tooth. Patency was checked with a #10 K-file (Dentsply Maillefer, Ballaigues, Switzerland) until the tip was visible at the apices. Then, the working length (WL) was established by subtracting 0.5 mm from this measurement. The root canals were prepared up to F3 (0.3 mm, 0.09 taper) with the ProTaper System (Dentsply Maillefer, Ballaigues, Switzerland) according to the manufacturer’s instructions.

During instrumentation, the root canals were copiously irrigated with 10 mL 5.25% NaOCl. After instrumentation, the canals were irrigated with 10 mL of 17% Ethylenediaminetetraacetic acid (EDTA), followed by 3 mL of 5.25% sodium hypochlorite (NaOCl) for 1 min, followed by a final flush with 10 mL of deionized water. Irrigating solutions were delivered using a 27-gauge side-vented needle (Max-I-Probe; Dentsply Maillefer, Ballaigues, Switzerland) and sonically activated for 1 min using the Endoactivator system (Dentsply Maillefer, Ballaigues, Switzerland) with a 25/04 tip. The tip was placed at −2 mm from the WL. Root canals were then dried with paper points. Teeth were randomly divided into 4 equally experimental groups (n = 10) and 2 control groups according to the type of sealer and the obturation techniques.

### 2.3. Root Canal Obturation

The HiFlow and Bio-C sealers were placed in a disposable syringe. They were both labeled during the mixing procedure with 0.1% Rhodamine B dye (Sigma-Aldrich, St. Louis, MO, USA) to assess fluorescence for the confocal microscopy.

Four groups were randomly divided as follows:

In group 1 (B/SC, n = 10), Bio-C sealer (Angelus, Londrína, PR, Brazil) was labeled with 0.1% Rhodamine B dye (Sigma-Aldrich, St. Louis, MO, USA) to assess fluorescence for the confocal microscopy. Bio-C sealer was delivered in the canals with a size 30 lentulo spiral (Dentsply Maillefer, Ballaigues, Switzerland). An F3 gutta-percha cone was then slightly coated with 20 µL of sealer mixture and slowly inserted into the WL. The cone was cut at the orifice with the heat carrier.

In group 2 (B/WVC, n = 10), the cone was placed as previously described (group 1) then packed down using System B Pluggers (0.06) (Sybron Endodontics, Orange, CA, USA) to 4 mm from the apex at 200 °C for 10 s. Canals were backfilled using an Obtura II (Obtura Spartan, Fenton, MO, USA).

In group 3 (H/SC, n = 10) and group 4 (H/WVC, n = 10), teeth were obturated with the same procedure but with the HiFlow (Brasseler USA^®^, Savannah, GA, USA) sealer using the SC and WVC techniques, respectively. A temporary filling material (Cavit, 3 M; ESPE, St. Paul, MN, USA) was placed coronally in all the specimens. Teeth were stored in a 37 °C incubator at 100% humidity for 2 weeks for complete setting. Negative control groups (n = 2) were filled with either HiFlow sealer or Bio-C without the fluorescent agent. Positive controls (n = 2) were left unobturated.

### 2.4. Sectioning of Roots and Preparation of Root Surfaces

Teeth were vertically embedded in an orthodontic resin block. They were sliced perpendicular to their long axis using slow speed diamond disks (25,000 rpm) under continuous water cooling at levels of 1 and 5 mm from the apex. Two slices of 2 mm thickness were obtained from each tooth. Apical and middle portions were polished with abrasive papers (500, 700, and 1200) to eliminate the debris from the cutting process. Sections were placed in an ultrasonic bath for 1 min at 45 °C and were mounted on glass slides.

### 2.5. Confocal Laser Analysis

Each section was examined under CLSM (10× magnification) (Zeiss LSM 710, Wetzlar, Germany). The emission wavelength was set at 561 nm. Digital images were analyzed with the software Image J (ImageJ software, NIH) to measure the maximum sealer penetration depths (μm) in the dentinal tubules at 4 circumferential points (12, 3, 6, and 9 o’clock). The tool “distance” was applied from the root canal surface to the deepest extent of the visible sealer. Measurements were performed by 1 observer and repeated 2 times to ensure reliability.

### 2.6. Statistical Analysis

The normality of the distribution was analyzed using the Kolmogorov–Smirnov test. The significance level was set at *p* ≤ 0.05 and the confidence interval at 95%. The Kruskal–Wallis test was used within groups to compare differences between middle and apical portions. The nonparametric Mann–Whitney U test was used for pairwise comparison between the type of sealers and the filling techniques (*p* < 0.05). Data statistical analysis was conducted by using SPSS 16.0 software (Chicago, IL, USA).

## 3. Results

### 3.1. Comparison between Cuts at 1 mm and 5 mm from the Apex in Each Group

The Kruskal–Wallis test showed that there was a statistical difference between the four groups at 1 mm from the apex (*p* = 0.0116). The mean penetration was more variable between the groups. However, similar statistical results were observed at 5 mm (*p* = 0.2026). Moreover, the sealer penetrated deeper at the −5 mm level compared with the −1 mm level in the four experimental groups (results shown in Table 1).

### 3.2. Comparison between the Sealers (HiFlow/BioC) Regardless of the Technique Used

Both sealers showed no statistically significant difference for the maximum diffusion at 1 mm (*p* = 0.7455) and 5 mm (*p* = 0.7251).

### 3.3. Comparison between the Obturation Techniques (SC/WVC) Regardless of the Sealer Used

**Figure 1:**
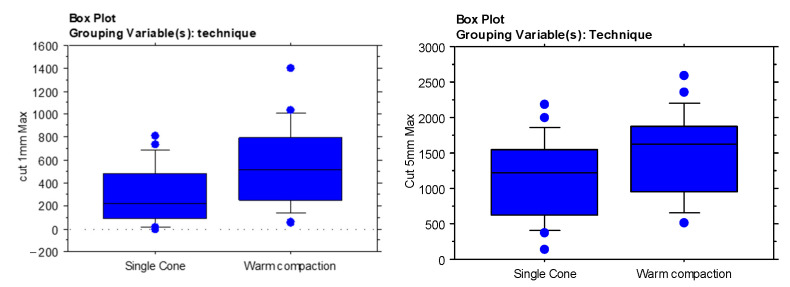
Box plot representation of the sealers penetration depth at 1 mm (left) and 5 mm (right) depending on the obturation techniques.

The WVC technique allowed for a better diffusion at 1 mm (*p* = 0.011) and at 5 mm from the apex (*p* = 0.034) than the SC (results shown in Table 2 and Figure 1).

## 4. Discussion

Many microorganisms persist in ramifications and isthmuses despite proper chemo-mechanical preparation. Therefore, the penetration of a sealer is required for the elimination of residual bacteria and biofilms sheltering into these anatomies [13]. The sealer’s diffusion in the tubules should be optimal to also obtain a hermetic seal and improve retention for a better long-term outcome [14]. Nevertheless, it is affected by various factors such as the physical and chemical properties of the sealer, the effectiveness of the removal of the smear layer, the anatomy of the root canal system, and the filling technique [15]. Moreover, the fine particles of the calcium silicate-based sealers (<1 μm) represent one of the major reasons why their deep diffusion is more likely to occur even with the SC technique, in addition to their basic pH which denatures the collagen fibers, their high flow rate, and their volume expansion of 0.2% with the setting results in tubular penetration [13,16].

It has been reported that the flushing effect and hydrodynamic agitation might affect the irrigation solutions’ efficiency and the smear layer removal [17]. In fact, its adherence forms physical barriers and contamination in the dentinal tubules, blocking the penetration of the sealer [18]. Therefore, the irrigation protocol provided in this study was characterized by the use of EDTA and sonic activation with Endoactivator [19].

CLSM was used to assess the diffusion; measures were taken with a method similar to that used by Bitter et al. [20]. Different techniques were proposed in the literature, like the use of scanning electron microscopy (SEM), optical microscopy, transmission electron microscopes (TEM), and stereomicroscopy. CLSM was chosen over all other techniques as sections are visualized at different levels, creating a 3D image. Moreover, no dehydration or gold coatings were required for specimen preparation. The integrity of the dentin was later preserved [18]. This method, unlike SEM, offers a wide and detailed vision without artifacts [21,22]. Previous studies showed that leaching of the fluorescent Rhodamine B was not possible. The very limited quantity (0.1%) used did not alter the sealer’s properties [21]. However, another agent, Fluo-3, was also used in a previous study by Jeong et al. An average penetration depth ranging between 200 and 400 μm was found [15], while others visualized a depth of up to approximately 1500 μm [21]. This difference was explained by the use of Rhodamine B, which was capable of leaching out and modifying the results [15]. In our study, no diffusion of this agent was noted. Rhodamine B could be suitable with the calcium silicate-based sealers. In addition, the complexity of the canal system might also interfere with the measurements. The oval shaped canals had a very challenging anatomy and should be taken into account in the selection of the specimen. The butterfly effect described by Russell was more likely to be seen in these configurations. Greater penetration was observed bucco-lingually than mesio-distally in some sections. This might explain the wide range of diffusion found with both sealers in our research as well as in various previous studies [15,21,23].

BC sealer is typically recommended with the single cone technique because heat might deteriorate its physical properties by decreasing the bond strength. The setting time and flow rate were reduced [24]. However, Heran et al. showed that calcium silicate-based sealers were not influenced by heat [25], whereas Fernandez et al. described filling more of the lateral canals with WVC [26]. Celikten et al. indicated that EndoSequence BC sealer had similar significant results in the number of voids and gaps, regardless of the three different obturation techniques applied [27].

The use of one tapered master cone matched better with the canal anatomy, which allowed similar obturation quality to WVC according to Alshehri et al. [28]. Some studies reported a predominance of one method over the other, while others advocated no significant difference between the techniques. No clear consensus has been reached indicating better tightness with one method over the other [21]. In fact, the major difference between the techniques is that endodontic sealer is mainly filled into the irregularities with the SC technique, whereas thermoplastified gutta-percha penetrates more completely in these areas with WVC [22].

**Figure 2:**
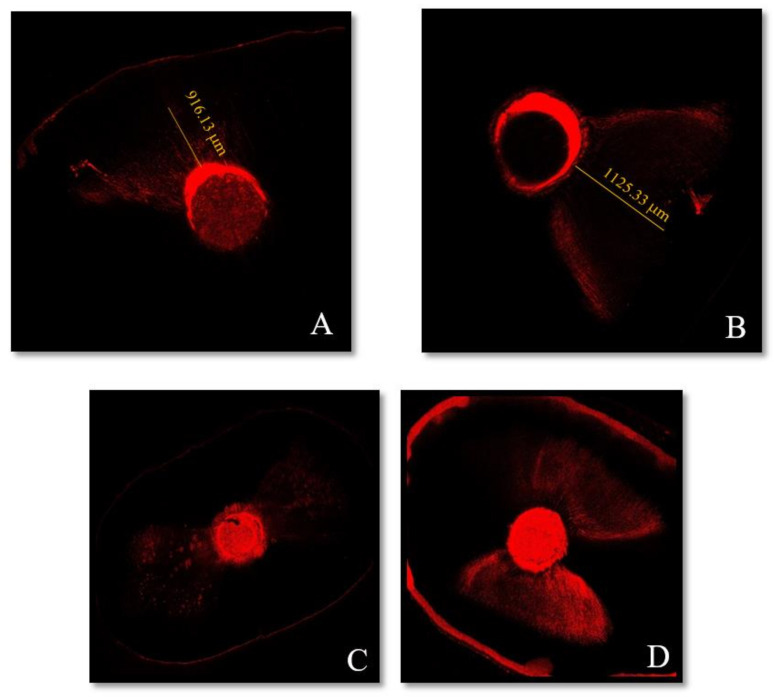
Representative confocal microscopic images of each sealer’s depth penetration in the dentinal tubules at 1 mm from the apex: (**A**) HiFlow sealer with the single cone (SC) technique and (**B**) HiFlow sealer with warm vertical compaction (WVC). Moreover, at 5 mm from the apex: (**C**) HiFlow sealer with the SC and (**D**) HiFlow sealer with WVC.

**Figure 3:**
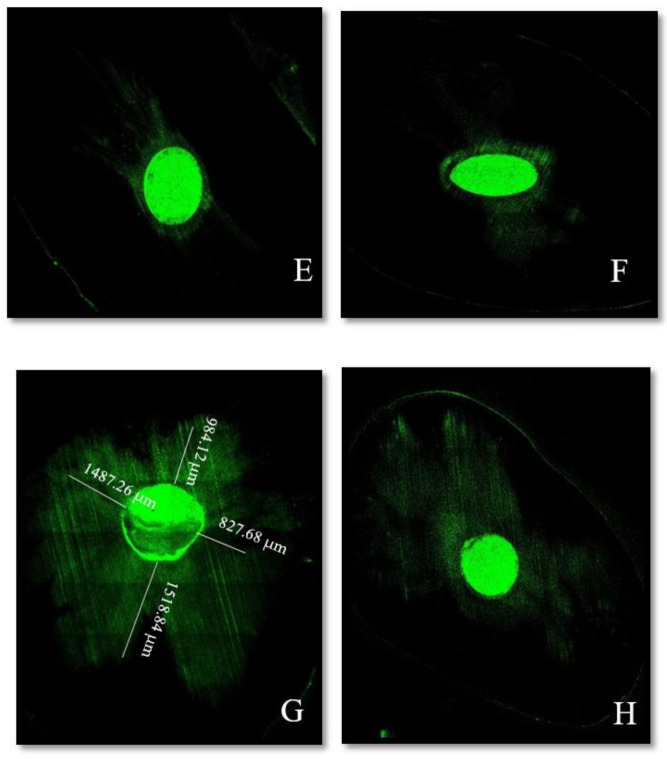
Representative confocal microscopic images of each sealer’s depth penetration in the dentinal tubules at 1 mm from the apex: (**E**) Bio-C sealer with the SC technique and (**F**) Bio-C sealer with WVC. Moreover, at 5 mm from the apex: (**G**) Bio-C sealer with the SC and (**H**) Bio-C sealer with WVC.

Concerning the epoxy resin sealer AH Plus (Dentsply), it was reported that heat affects its properties [11,12,20]. Therefore, it was not exploited in these conditions in our study. Wang Y et al. found similar results with the iRoot SP using the two obturation techniques. They explained that heat had not shown an impact on the apical third [29]. McMichael et al. found similar tubule penetration of Endosequence BC with both single cone and warm vertical compaction at both levels [21]. However, the results in our study showed deeper penetration with WVC (Figure 2B,D). This difference might be related to the greater compressive forces applied coronally during obturation which would improve the sealer’s penetration in the apical third (Figure 3). Maximum measurements were also observed in the middle portion for both sealers regardless of the filling technique (*p* > 0.7). The significant difference can be attributed to the increase in the tubules’ density and diameter in the coronal direction. Moreover, the sclerotic dentin and the hardness of the smear layer in the apical third might create a physical barrier to the sealer’s penetration [27].

The new modified tricalcium silicate sealers could still be promising even when thermoplastic techniques such as WVC are used, resulting in an improvement in the quality of the filling. Therefore, the best obturation technique for this material is still a matter of debate. However, despite the temperature of the devices being set at 200 °C, the true temperature generated by most heat carriers appears to be much lower [30].

Parameters such as physicochemical properties, cellular responses, and long-term clinical considerations should be investigated further.

Nevertheless, some authors showed that retreatment techniques were not able to fully remove BC sealers [14]. Further investigations are needed concerning their retreatment.

The null hypothesis of this study was rejected: the application of heat using WVC enhanced the calcium silicate-based sealer penetration in the dentinal tubules. No differences were observed comparing the HiFlow with the Bio-C sealer. Although the BC sealers are recommended with the SC technique, it might be interesting to reconsider the application of Schilder’s principles with these newly introduced sealers.

## Figures and Tables

**Table 1:** Mean penetration depth (µm) of two calcium silicate-based sealers.

	Group	Bio-C-SC	Bio-C-WVC	HiFlow-SC	HiFlow-WVC	Sig
Level						
1 mm		397.428 µm ± 77.46	447.076 µm ± 303.082	194.24 µm ± 227.369	672.82 µm ± 390.807	0.0116
5 mm		1080.92 µm ± 575.228	1421.98 µm ± 509.75	1115.051 µm ± 619.506	1567.634 µm ± 666.873	0.2026 *
Sig		0.0065	0.0007	0.0007	0.0052	

* Analysis of variance: no statistically significant difference among the mean maximum depth measurements.

**Table 2:** Penetration depth (µm) according to the obturation techniques at different levels (1 and 5 mm).

	Level	Single Cone	Warm Vertical Compaction	Sig
Obturation Technique				
1 mm		295.776 µm ± 252.568	559.488 µm ± 359.539	0.011
5 mm		1097 µm ± 582.119	1494.457 µm ± 582.511	0.0349

## Data Availability

The data presented in this study are available on request from the corresponding author.
